# AP39 alleviates HHCY-induced myocardial remodeling by regulating FUNDC1-mediated mitochondrial dynamics via S-sulfhydration of NEDD8/CUL4B

**DOI:** 10.3389/fphar.2026.1729145

**Published:** 2026-03-09

**Authors:** Yaling Li, Jianghe Jiang, Yingchun Song, Ting Yang, Xinghua Yuan, Xuan Li, Chenlong Luo, Yan Shi, Shengquan Liu, Chun Chu, Jun Yang

**Affiliations:** 1 Department of Cardiology, The First Affiliated Hospital, Hengyang Medical School, University of South China, Hengyang, Hunan, China; 2 Department of Pharmacy, The Second Affiliated Hospital, Hengyang Medical School, University of South China, Hengyang, Hunan, China; 3 Hunan Provincial Key Laboratory of Multi-omics and Artificial Intelligence of Cardiovascular Diseases, University of South China, Hengyang, Hunan, China; 4 Clinical Research Center for Myocardial Injury in Hunan, Hengyang, Hunan, China

**Keywords:** AP39, hyperhomocysteinemia, mitochondrial dynamics, myocardial remodeling, NEDD8/CUL4B pathway

## Abstract

**Introduction:**

Hyperhomocysteinemia (HHCY) is a well-recognized risk factor for cardiovascular diseases; however, the molecular mechanisms underlying HHCY-induced myocardial remodeling remain unclear. This study aimed to investigate the role of mitochondrial dysfunction and cardiomyocyte senescence in HHCY-associated myocardial remodeling and to explore the potential protective effects of AP39, a mitochondria-targeted hydrogen sulfide (H_2_S) donor.

**Methods:**

An integrated approach combining retrospective clinical analysis, animal models, and cellular experiments was employed. Associations between homocysteine (HCY) levels and left ventricular hypertrophy were analyzed in hypertensive patients. In vivo and in vitro models of HHCY were used to assess cardiac function, myocardial fibrosis, cellular senescence, mitochondrial dynamics, and underlying molecular mechanisms, with or without AP39 intervention.

**Results:**

Clinical analysis demonstrated that HHCY was significantly associated with left ventricular hypertrophy, and elevated HCY levels increased the risk of ventricular hypertrophy. In animal models, HHCY resulted in impaired cardiac function, evidenced by reduced left ventricular fractional shortening and increased left ventricular end-systolic diameter, accompanied by myocardial fibrosis and cardiomyocyte senescence. AP39 treatment markedly ameliorated these pathological changes. Mechanistically, AP39-derived H_2_S promoted S-sulfhydration of the NEDD8/CUL4B complex, thereby reducing ubiquitin-dependent degradation of FUNDC1. Upregulation of FUNDC1 restored mitochondrial dynamic homeostasis by weakening its interaction with DRP1, ultimately suppressing cardiomyocyte senescence.

**Discussion:**

These findings uncover a previously unrecognized mechanism by which AP39 preserves mitochondrial homeostasis through regulation of the FUNDC1–DRP1 axis via NEDD8/CUL4B-dependent S-sulfhydration. This study identifies a novel therapeutic target and provides mechanistic insight into HHCY-associated myocardial remodeling.

## 1 Introduction

Homocysteine (HCY) is a sulfur-containing amino acid and an intermediate product of methionine metabolism in the body. Hyperhomocysteinemia (HHCY) is diagnosed when the plasma HCY level is ≥ 15.0 μmol/L (Moretti and Caruso, 2019). A study by Sun et al. reported that plasma HCY levels are strongly associated with the incidence of cardiovascular diseases and mortality (Sun et al., 2009). Meanwhile, HHCY can induce pathological myocardial remodeling (Cao et al., 2021; Zheng et al., 2019). Emerging studies have identified cardiomyocyte senescence as a key mechanism contributing to myocardial remodeling (Liu et al., 2025). Annoni et al. revealed a marked increase in the expression of type I and type III collagen in the myocardium of senescent rats (Annoni et al., 1998). A recent transgenic mouse study further confirmed that specific deletion of the senescence marker p16 in cardiomyocytes significantly reduces post-infarction scar size and improves cardiac function, proving that senescent cardiomyocytes are major drivers of pathological remodeling (Redgrave et al., 2023). These findings collectively underscore the pivotal role of cellular senescence in the onset and progression of myocardial fibrosis (Kwak et al., 2015). Notably, HHCY can induce cellular senescence by activating galactokinase (Sun et al., 2019; Kovalska et al., 2018). Recent studies have further found that HHCY also inhibits thioredoxin expression and activates the p38 MAPK pathway to promote myocardial ROS production, thereby accelerating pathological senescence of cardiomyocytes and exacerbating fibrosis (Goyal et al., 2024). Taken together, the current evidence suggests that targeting cardiomyocyte senescence may represent a promising therapeutic strategy for preventing or mitigating HHCY-induced myocardial remodeling.

Mitochondrial dynamics refers to the continuous fusion and fission processes that maintain mitochondrial homeostasis (Vincow et al., 2013; Durcan et al., 2014). Recent reviews further clarify that this balance is essential for preserving mitochondrial cristae organization and oxidative phosphorylation efficiency, and its disruption in the aging heart directly leads to structural damage and functional decline of the myocardium (Patyal et al., 2025). Song et al. demonstrated that genetic knockout of either dynamin-related protein 1 (DRP1) or mitofusin 1/2 (MFN1/2) results in severe cardiac abnormalities, highlighting the essential role of mitochondrial dynamics in cardiac physiology (Song et al., 2015). Scheckhuber et al. first reported in *Nature Cell Biology* that the DRP1 gene is linked to cellular senescence, establishing mitochondrial dynamics as a novel regulatory pathway of senescence (Scheckhuber et al., 2007). Consistent with this concept, Faitg J et al. observed upregulated DRP1 and downregulated MFN2 in senescent tissues (Faitg et al., 2019). Recent studies further identified that DRP1 phosphorylation at Ser616 and enhanced ubiquitination of MFN2 are key molecular events driving this imbalance, which in turn activates ROS-dependent senescence pathways in cardiomyocytes (Uchikado et al., 2022). Moreover, Tsushima et al. demonstrated a mouse model of obesity-induced lipotoxic injury that modulating mitochondrial fission–fusion balance alleviates myocardial remodeling, further underscoring the importance of dynamic mitochondrial regulation in cardiac homeostasis (Tsushima et al., 2018). Collectively, these findings raise an important question: does dysregulated mitochondrial dynamics contribute to cardiomyocyte senescence and HHCY-induced myocardial remodeling?

FUN14 domain-containing protein 1 (FUNDC1) is a novel mitochondrial receptor that regulates mitochondrial turnover through direct interaction with LC3 (Kuang et al., 2016). Studies by the Wu and Chen research teams have demonstrated that under hypoxia or mitochondrial stress, phosphorylation of FUNDC1 at Ser17 activates FUNDC1 and initiates mitophagy, whereas FUNDC1 deletion markedly suppresses hypoxia-induced mitophagy (Wu et al., 2014; Chen et al., 2014). In addition, mitochondria-localized DRP1 and fragmented mitochondria are preferentially cleared through FUNDC1-dependent mitochondrial degradation (Wang et al., 2020). Beyond its role in mitophagy, Ding et al. further identified that FUNDC1 directly interacts with DRP1 (Bai et al., 2023). These findings suggest that FUNDC1 serves as a central regulator linking mitophagy to mitochondrial dynamics and may represent a promising therapeutic target for suppressing cellular senescence and preventing myocardial remodeling. Neural precursor cell-expressed developmentally downregulated protein 8 (NEDD8) is a ubiquitin-like molecule that modifies target proteins through covalent conjugation (neddylation) (Zhang et al., 2024). Cullin 4B (CUL4B), a member of the Cullin family, forms the core of the CUL4B-RING ubiquitin ligase complex (Petroski and Deshaies, 2005). Neddylation of CUL4B by NEDD8 is essential for maintaining complex activity, thereby mediating the ubiquitin-dependent degradation of substrate proteins and regulating diverse biological processes, including cell cycle progression and intracellular signaling (Sarikas et al., 2011). Increasing evidence indicates that NEDD8 and CUL4B are closely involved in cardiovascular disease pathogenesis (Ouyang et al., 2022). For instance, Su et al. showed that NEDD8 promotes cardiomyocyte proliferation and myofibrillogenesis via Cullin regulation (Littlejohn et al., 2024), while NEDD8 inhibition attenuates doxorubicin-induced myocardial fibrosis (Chen K. H. et al., 2024). Beyond protein degradation, neddylation also influences mitochondrial energy metabolism and quality control, as suppression of neddylation alters mitochondrial morphology and reprograms cellular metabolism (Zhou et al., 2019). Given the central role of mitochondrial dysfunction is crucial in metabolic cardiovascular diseases and myocardial remodeling, it is plausible that NEDD8-mediated CUL4B activation contributes to HHCY-induced myocardial remodeling by disturbing mitochondrial dysfunction, although the precise mechanisms remain unclear.

Hydrogen sulfide (H_2_S), the third gasotransmitter, exerts well-established cardioprotective effects. Penget al. reported that H_2_S prevent cellular senescence via mitochondria regulation (Chen T. et al., 2024), while Liuet al. demonstrated that H_2_S prevents fibrosis by delaying premature cellular senescence (Wang et al., 2024). However, conventional H_2_S donors such as NaHS are inorganic salts with limited stability. AP39, a mitochondria-targeted H_2_S donor, effectively increases mitochondrial H_2_S levels and reduces endothelial senescence (Zhao et al., 2016; Karwi et al., 2017; Latorre et al., 2018). Furthermore, accumulating studies indicate that the cardioprotective actions of H_2_S largely depend on protein S-sulfhydration, a post-translational modification essential for mitochondrial and cardiac homeostasis (Wu et al., 2018; Bibli et al., 2019; Meng et al., 2018; Cheung and Lau, 2018; Peng et al., 2023). Notably, H_2_S mediates S-sulfhydration of Miro2 (a key mitochondrial dynamics regulator) at cysteine residues C185/C504, which maintains mitochondrial network integrity and promotes cell survival (Feng et al., 2024). Nevertheless, whether H_2_S ameliorate HHCY-induced myocardial remodeling by modulating mitochondrial dynamics through S-sulfhydration remains unknown and warrants further investigation.

In summary, this study aims to address the following key questions: (1) Does HHCY induce alterations in mitochondrial dynamics that contribute to cardiomyocyte senescence and myocardial remodeling? (2) Can AP39 ameliorate mitochondrial fission/fusion by regulating the interaction between FUNDC1 and DRP1? (3) How do the NEDD8/CUL4B pathway and H_2_S exert protective effects by interfering with mitochondrial function? The results of this study may provide a novel intervention target for HHCY-related myocardial remodeling.

## 2 Materials and experimental methods

### 2.1 Western blotting and immunofluorescence

All primary antibodies used for Western blotting (WB) and immunofluorescence (IF) were purchased from the corresponding commercial suppliers, and their detailed information is listed as follows: GAPDH (Proteintech, USA; 10494-1-AP; WB 1:8000), α-smooth muscle actin (α-SMA) (Proteintech, USA; 14395-1-AP; WB 1:4000), Collagen type III (Proteintech, USA; 22734-1-AP; WB 1:800), FUNDC1 (Proteintech, China; AWA51295; IF 1:100, WB 1:1000), LC3B (Proteintech, USA; 14600-1-AP; IF 1:500, WB 1:4000), MFN1 (Proteintech, USA; 13798-1-AP; WB 1:5000), MFN2 (Proteintech, USA; 12186-1-AP; WB 1:20000), DRP1 (Proteintech, USA; 12957-1-AP; WB 1:5000), P53 (Proteintech, USA; 10442-1-AP; WB 1:20000), P16 (Proteintech, USA; 10883-1-AP; WB 1:3000), NEDD8 (Abways, China; AWA11003; WB 1:1000), and CUL4B (Abways, China; AWA48229; WB 1:1000). For secondary antibodies, HRP-conjugated goat anti-mouse/goat anti-rabbit IgG (Proteintech, USA; SA00001-2; 1:5000) was used for Western blot detection. Cy3-conjugated goat anti-mouse IgG (H + L) (Proteintech, USA; SA00001-2; 1:10000) and FITC-conjugated goat anti-rabbit IgG (H + L) (Beyotime, China; A0562; 1:500) were used for immunofluorescence staining.

### 2.2 Culture and treatment of H9c2 cardiomyocytes

The H9c2 cell line was obtained from the Cell Bank of the Chinese Academy of Sciences (Shanghai, China). Cells were cultured in Dulbecco’s Modified Eagle Medium (DMEM) supplemented with 10% calf serum (Procell, Cat. No.: 164210) and maintained in a humidified incubator at 37 °C with 5% CO_2_. H9c2 cells were randomly seeded into 6-well culture plates at an appropriate density, and treated as described below. Cardiomyocyte injury was induced with 1 mM HCY. The experimental groups were set as follows: Control group: H9c2 cells cultured under standard conditions (37 °C, 5% CO_2_) for 24 h. HHCY group (1 mmol/L): Cells incubated with 1 mM HCY for 24 h H_2_S intervention group (HHCY + AP39): Cells pretreated with AP39 (100 nM) prior to the addition of diluted HCY and cultured for 24 h. DL-propargylglycine group (PAG group): Cells pretreated with AP39 (100 nM),followedby incubation with PAG (2 mmol/L (Pan et al., 2011)); After 30 min, 1 mM HCY was added and cells were cultured for 24 h. DMSO group: Cells cultured with an equivalent volume of DMSO for 24 h under standard conditions. AP39 group: Cells incubated with AP39 (100 nM) for 24 h under standard conditions.

### 2.3 Detection of mitochondrial H_2_S (Mito-HS fluorescent probe)

Mitochondrial H_2_S levels were assessed using the Mito-HS fluorescent probe as described by Li et al. (Fang et al., 2020). After treatment, H9c2 cardiomyocytes cultured in 6-well plates were washed three times with phosphate-buffered saline (PBS)and incubated with 10 μM Mito-HS at 37 °C for 1 h. The probe was then removed, and cells were washed three additional times with PBS. Fluorescence was subsequently examined and imaged using a microscope (OLYMPUS, Japan).

### 2.4 Detection of cellular senescence (β-galactosidase (SPIDER-βGal) staining)

Cellular senescence was evaluated using the SPIDER-βGal staining kit. After treatment, H9c2 cells in 6-well plates were washed three times with PBS and fixed with 4% formaldehyde (EMD Millipore, USA) for 10 min at room temperature. Following three additional PBS washes, the SPIDER-βGal working solution (Dojindo, Japan), and cells were incubated at 37 °C for 30 min. Senescence-associated fluorescence was then visualized using an OLYMPUS microscope (Japan).

### 2.5 Detection of mitochondrial membrane potential (JC-1 staining)

Mitochondrial membrane potential was assessed using the JC-1 assay. After treatment, H9c2 cells in 6-well plates were washed once with PBS. For each well, 1 mL of cell culture medium and 1 mL of JC-1 staining working solution were added, and cells were returned to the incubator for 20 min. During the incubation, 1× JC-1 staining buffer was prepared by diluting 1 mL of 5× JC-1 staining buffer with 4 mL of distilled water, which was stored on ice for later use. After incubation, H9c2 cardiomyocytes in 6-well plates were washed twice with the prepared 1× JC-1 staining buffer, 2 mL of cell culture medium was added, and changes in mitochondrial membrane potential were observed under a microscope (OLYMPUS Corporation, Japan).

### 2.6 RNA sequencing (RNA-seq)

RNA-seq was conducted by Shanghai Gene Chem Co., Ltd. Total RNA was extracted from treated H9c2 cardiomyocytes, and RNA integrity and purity were evaluated prior to library construction. Sequencing libraries were generated and subjected to standard quality control procedures. High-throughput sequencing was then performed, and raw reads were processed to obtain clean sequence data. Differential gene expression and functional enrichment analyses were subsequently carried out to identify transcriptional alterations associated with the experimental treatments.

### 2.7 Immunofluorescence

Immunofluorescence staining on cell-coated slides was performed to assess FUNDC1-dependent mitophagy under different treatment conditions. H9c2 cardiomyocytes stored at −80 °C were thawed and cultured in a humidified incubator. When cells reached approximately 30% confluence, they were seeded into 6-well plates containing sterilized glass slides. Upon reaching ∼50%, cells were subjected to the designated treatments for 24 h. Following treatment, cell was fixed with 4% paraformaldehyde for 1 h and permeabilized with 0.5% Triton X-100 for 20 min. After blocking at room temperature for 30 min, cells were incubated with appropriately diluted primary antibodies overnight at 4 °C. Fluorescent secondary antibodies were added in a dim environment to prevent photobleaching, followed by incubation at 37 °C for 1 h in a humid chamber. Coverslips were mounted with DAPI-containing antifade reagent and stored at 4 °C. Fluorescence colocalization was visualized using a fluorescence microscope.

### 2.8 SiRNA interference

According to the product manual, siRNAs targeting FUNDC1 and negative control siRNAs (Sangon Biotech, Shanghai, China) were transiently transfected into H9c2 cells using Lipofectamine 3000 transfection reagent (Life Technologies, USA). The siRNA sequences were as follows.

Fundc1-001: AGA​GCG​ATG​ACG​AGT​CTT​A
Fundc1-002: GCA​CCT​GAA​ATC​AAC​AAT​A
Fundc1-003: TTC​CGG​ACC​TAT​GGT​AGA​A

At 48 h post-transfection, protein levels were examined by WB, and the siRNA exhibiting the greatest knockdown efficiency was selected for subsequent experiments.

### 2.9 Co-immunoprecipitation (CO-IP)

Following the designated transfection, cells were lysed in ice-cold cell lysis buffer (Beyotime, Cat. No.: P0013D) supplemented with phosphatase inhibitor, a protease inhibitor cocktail (Thermo Fisher Scientific, Cat. No.: #78446), and DTT (Thermo Fisher Scientific, Cat. No.: #R0861). The lysate was centrifuged at 12,000×g for 10 min at 4 °C, and a portion of the supernatant was collected as the Input group. The remaining supernatant was incubated with control IgG and anti-Protein A/G magnetic beads (Lingen Bio-tech, Cat. No.: L-1004) under gentle rotation overnight at 4 °C. Subsequently, fresh Protein A/G magnetic beads were added and incubated for an additional 4 h at room temperature. The beads were washed three times with TBST buffer and eluted by boiling in 1×sample loading buffer (Beyotime, Cat. No.: #P0015A) for 10 min. The immunoprecipitated proteins were then subjected to SDS-PAGE and analyzed by WB.

### 2.10 Biotin modification assay

Treated H9c2 cells were lysed in 300 μL of HENS buffer and collected into 1.5 mL microcentrifuge tubes. Cells were sonicated (4 cycles: 10 s of sonication followed by 10 s of interval) using an ultrasonic homogenizer, then centrifuged at 12,000×g for 15 min at 4 °C. The supernatant was transferred to a 15 mL tube. and protein concentration was quantified using the BCA assay. Aliquots containing no more than 800 μg of total protein were adjusted to 1 mL with HEN buffer, followed by the addition of 40 μ L of pre-cooled 10% CHAPS (4 °C) was added, followed by thorough vortex mixing. Next, 4 mL of blocking solution was added, mixed andincubated in a 50 °C for 20 min. Ten milliliters of pre-chilled acetone (−20 °C) were added gradually with mixing, and samples were incubated at −20 °C for 20 min. After centrifugation at 4,000×g for 10 min at 4 °C, the supernatant was discarded. The protein pellet was resuspended in 80 μL of HENS buffer and transferred to a 1.5 mL microcentrifuge tube. Subsequently, 4 μ L of biotin-HPDP and 0.8 μ L of sodium ascorbate were added, mixed, and incubated at 25 °C for 2 h. Protein was precipitated again by adding 250 μ L of pre-chilled acetone, followed by incubation at −20 °C for 20 min and centrifugation at 4,000×g for 10 min. After removing the supernatant and briefly rinsing the pellet with cold acetone, the protein was resuspended in 80 μ L of HENS buffer. Neutralization buffer (160 μ L) and streptavidin–agarose beads (20 μ L) were added, and samples were rotated overnight at 4 °C.The next day, samples were washed five times with 600 μ L of 600 mM NaCl, centrifuging at 500×g for 30 s between washes. Bound proteins were eluted with 20 μ L of elution buffer at 37 °C for 20 min, mixed with 10 μ L of 5×SDS-PAGE loading buffer, centrifuged, and boiled at 100 °C for 5 min. Biotin-modified proteins were subsequently analyzed by WB.

### 2.11 Establishment and grouping of animal models

Fifty 8-week-old male Sprague-Dawley (SD) rats (weighing: 0.25–0.3 kg) were obtained from the Animal Department of University of South China. Animals were housed under controlled environmental conditions (24 °C ± 2 °C, adequate ventilation, free access to food and water, and a 12-h light/dark cycle), After 1 week of acclimatization, rats were randomly assigned into five groups (n = 10 per group):Control group: Normal drinking water; HHCY group: Drinking water supplemented with L-methionine (10 g/L) (Ungvari et al., 2003; de Rezende and D'Almeida, 2014); HHCY + AP39 group (AP39 intervention): Drinking water containing L-methionine (10 g/L) + daily intraperitoneal AP39 (56 μmol/kg); HHCY + AP39 + PAG group (PAG group): Drinking water containing L-methionine (10 g/L) + daily intraperitoneal AP39 (56 μmol/kg) + PAG (40 mg/kg); AP39 group: Normal drinking water + daily intraperitoneal AP39 (56 μmol/kg).

Drinking water for the HHCY and HHCY + AP39 groups was replaced daily to maintain effective concentration. After 10 weeks of intervention, serum HCY levels were measured to confirm successful establishment of the HHCY model. Subsequently, rats in the AP39 group and HHCY + AP39 group continued to received AP39 (56 μ mol/kg) for an additional 4 weeks; the PAG group received AP39 (56 μ mol/kg) plus PAG (40 mg/kg) for 4 weeks. The Control and HHCY groups were administered an equivalent volume of normal saline during the same period.

### 2.12 Echocardiography and calculation of cardiac index

Four weeks after AP39 intervention, SD rats were weighed and anesthetized with sodium pentobarbital (50 mg/kg, i. p.) after removal of chest hair. M-mode transthoracic echocardiography was performed with rats in the supine position. in the supine position. The following parameters were recorded: left ventricular posterior wall thickness at diastole (LVPWD), left ventricular end-diastolic diameter (LVDD), and left ventricular end-systolic diameter (LVSD). Left ventricular fractional shortening (LVFS) was calculated to assess cardiac function. Following echocardiography, rats were euthanized by an overdose of sodium pentobarbital (150 mg/kg, i. p.). Body weight was measured, and the heart was excised, rinsed with isotonic saline, blotted dry, and weighed. The cardiac weight index was then calculated. All experimental procedures were approved by the Ethics Committee of University of South China (Approval No.: SYXK (Xiang) 2024-0027).

### 2.13 Masson staining

Heart tissues from SD rats in each group were fixed in 4% formaldehyde for 24 h and subsequently embedded in paraffin. The tissues were sectioned into 5 μ m slices and subjected to baking, deparaffinization, and staining, followed by rinsing with tap water. The sections were then immersed sequentially in distilled water and weakly alkaline solvents (e.g., PBS or ammonia hydroxide). After removing excess moisture from the slides, and subsequent steps (staining, rinsing, differentiation, counterstaining) were performed. After washing, slices were observed under a light microscope.

### 2.14 Collection of clinical data

A total of 4,355 patients with essential hypertension who were hospitalized at the First Affiliated Hospital of University of South China from January 2019 to January 2022 were retrospectively enrolled from the electronic medical record database. Among them, 1,264 patients had complete data, including 1,017 patients with elevated serum HCY (accounting for 80.46%) and 247 patients without elevated HCY (accounting for 19.54%), with an age range of 40–80 years. Patients were excluded if they had secondary hypertension, severe valvular heart disease (moderate-to-severe valvular regurgitation), dilated cardiomyopathy, hypertrophic obstructive cardiomyopathy, malignant tumors, hematological diseases, or a history of oral folic acid or B vitamins (drugs affecting HCY metabolism) before or after admission. This study was approved by the Ethics Committee of the First Affiliated Hospital of University of South China (Ethics No.: 2022LL0128001), approval date: 28 January 2022). Patients’ personal information was strictly protected. All tissue samples analyzed in this study were obtained from patients undergoing standard clinical care procedures.

### 2.15 Statistical analysis

For clinical data, independent samples t-test was used to compare continuous variables between two groups. One-way analysis of variance (ANOVA) was applied for comparisons among multiple groups, followed by LSD-t test for pairwise comparisons; Chi-square (χ^2^) test was used for comparison of categorical data; Multivariate Logistic regression analysis was performed to calculate the odds ratios (ORs) and 95% confidence intervals (95% CIs) for disease risk across different HCY level groups. Receiver operating characteristic (ROC) curve analysis was conducted to assess the predictive value of serum HCY for left ventricular hypertrophy, including determination of the area under the curve (AUC), optimal cut-off value, and specificity. The statistical significance of the AUC was verified by Z-test.

Other experimental data were expressed as mean ± standard error of the mean (SEM). For comparison between two groups, unpaired two-tailed t-tests were used. For comparisons among more than two groups, one-way ANOVA followed by Bonferroni multiple comparison test was applied. For experiments involving two independent variables, two-way ANOVA followed by Bonferroni multiple comparison tests were used. Statistical analysis was performed using GraphPad Prism 8.2 (GraphPad Software, San Diego, CA, USA). A p-value <0.05 was considered statistically significant.

## 3 Results

### 3.1 Clinical observations: serum HCY level is significantly associated with ventricular hypertrophy and predicts its occurrence

To determine whether serum HCY level are associated with ventricular hypertrophy, we retrospectively analyzed data from 1,264 patients with essential hypertension who were hospitalized between January 2019 and January 2022 at the First Affiliated Hospital of the University of South China. Among these patients, 1,017 (80.46%) had elevated serum HCY levels, whereas 247 (19.54%) had normal levels.

**FIGURE 1:**
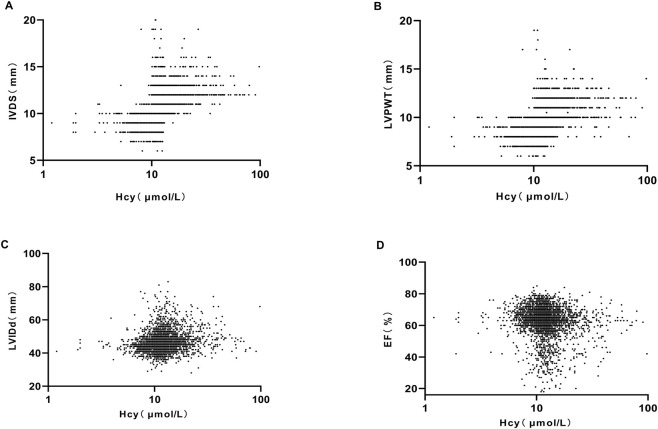
Correlations between serum HCY levels and left ventricular structure-function parameters. **(A)** Scatter plot of serum HCY vs. IVDS. **(B)** Correlation of serum HCY with left ventricular posterior wall thickness in diastole (LVPWT, mm). **(C)** Relationship between serum HCY and LVIDd. **(D)** Scatter plot of serum HCY vs. EF. left ventricular internal dimension in diastole: LVIDd; homocysteine: HCY; left ventricular ejection fraction: EF; interventricular septal dimension in diastole: IVDS.

Echocardiographic assessment revealed that, compared with the hypertension-only group, the hypertension + HHCY group showed significantly increased interventricular septal thickness (IVST), left ventricular end-diastolic diameter (LVEDD), left ventricular posterior wall thickness (LVPWT), and left ventricular ejection fraction (LVEF) (P < 0.05) (Supplementary Table S1; Figure 1). After adjusting for confounding factors using propensity score matching (PSM). The hypertension + HHCY group still exhibited significantly significantly greater IVST and LVPWT than the hypertension-only group (P < 0.05), whereas no significant differences were observed in LVEF or LVEDD (P > 0.05) (Supplementary Table S2). These findings indicate that the prevalence of ventricular hypertrophy is higher in patients with hypertension and HHCY compared with those with hypertension alone.

Based on the Expert Consensus on the Diagnosis and Treatment of HHCY, patients were stratified into four groups based on serum HCY levels: Q1 (normal): <10 μmol/L (n = 246); Q2 (mild elevation): 10–15 μmol/L (n = 534); Q3 (moderate elevation): 15–30 μmol/L (n = 414); Q4 (severe elevation): >30 μ mol/L (n = 61). The incidence of left ventricular hypertrophy (LVH) was 44.30% (109/246) in Q1, 53.04% (288/534) in the Q2, 50.00% (207/414) in Q3, and 62.30% (38/61) in Q4. Compared with the Q1, the risk of LVH was 1.42-fold higher in the Q2 (P < 0.05) and 1.90-fold higher in the Q4 (P < 0.05). The 1.263-fold increased risk in Q3 was not statistically significant (P > 0.05) (Table 1).

**TABLE 1:** The prevalence of left ventricular hypertrophy at different levels of HCY.

Variable	Number of patients	Prevalence of left ventricular hypertrophy (%)	Or (95%CI)	P-value
Q1	246	109 (44.30%)	1.000	—
Q2	543	288 (53.04%)	1.420 (1.049–1.922)	0.023
Q3	414	207 (50.00%)	1.263 (0.920–1.734)	0.149
Q4	61	38 (62.30%)	1.900 (1.126–3.518)	0.018

Q1, Hcy<10 mmol/L; Q2, Hcy10-15 mmol/L; Q3, Hcy15-30 mmol/L; Q4, Hcy>30mmolL; HCY, homocysteinemia.

To further evaluate the predictive value of serum HCY for LVH in hypertensive patients, ROC curve analysis was performed. Serum HCY demonstrated a significant predictive ability for LVH (P < 0.05), with an area under the curve (AUC) of 0.737 and a 95% confidence interval (95% CI: 0.722–0.751). The specificity was 0.752, indicating a relatively low false-positive rate. The optimal cut-off value for predicting LVH was determined as 12.85 μ mol/L, with a standard error (SE) of 0.007 (P < 0.001). This threshold may serve as a useful reference for clinical practice, suggesting that hypertensive patients with serum HCY levels exceeding this cut-off have an increased likelihood of developing LVH (Table 2).

**TABLE 2:** The predictive value of serum HCY levels for left ventricular hypertrophy in patients with hypertension.

Variable	Cut-off value	SE	P	AUC (95%CI)	Sensitivity	Specificity
HCY	12.85	0.007	P < 0.001	0.737 (0.722–0.751)	0.475	0.752

HCY, homocysteinemia.

### 3.2 HHCY induces myocardial remodeling in SD rats, and this process is antagonized by the H_2_S donor AP39

To further validate the association between HHCY and myocardial remodeling, an HHCY rat model was established by administering L-methionine (10 g/L) in drinking for 10 consecutive weeks. Approximately 40 uL of tail vein blood was collected. And the model was considered successful when plasma HCY levels reached ≥15.0 μmol/L (Supplementary Table S3). Echocardiographic analysis demonstrated that compared with the Control group, the HHCY group exhibited a significant reduction in LVFS (P < 0.05) and a significant increase LVESD (P < 0.05), indicating impaired cardiac function (Table 3).

**TABLE 3:** The levels of echocardiographic parameters in each group of rats (Mean ± SD).

Groups	LVESD (mm)	LVEDD (mm)	LVFS (%)	Number
Control	3.17 ± 0.62	6.05 ± 0.99	4.70 ± 0.98	10
HHCY	3.86 ± 0.72*	6.43 ± 0.61	4.10 ± 0.97*	8
HHCY + AP39	3.23 ± 0.36^#^	7.05 ± 0.47	5.41 ± 0.48^#^	8
PAG	4.18 ± 0.30^$^	6.65 ± 1.12	3.53 ± 0.76^$^	7
AP39	3.61 ± 0.60	7.23 ± 0.57	5.02 ± 0.68	10

*p < 0.05 vs. Control.

^#^p < 0.05 vs. HHCY.

^$^p < 0.05 vs. HHCY + AP39.

**FIGURE 2:**
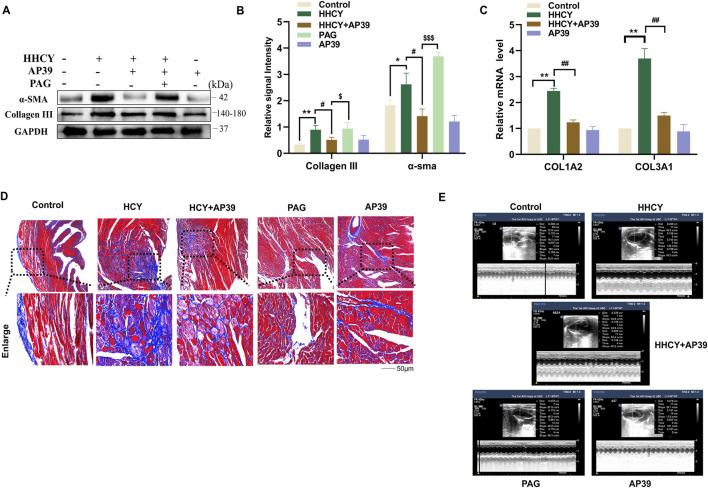
**(A,B)** Expression of α-SMA, collagen III in the myocardial tissues of SD rats induced by HHCY in each group. (*p < 0.05 vs. Control, **p < 0.01 vs. Control; ^#^p < 0.05 vs. HHCY; ^$$$^p < 0.005 vs. HHCY + AP39). **(C)** Relative expression of col1α2 and col3α1 genes in each group of SD rats as detected by QT-PCR. **(D)** Masson staining to detect the myocardial fiber conditions in the myocardial tissues of each group of SD rats (blue markers indicate myocardial fiber tissue). **(E)** Representative echocardiographic images of each group.

WB analysis showed that the protein levels of fibrosis markers α-SMA and Collagen III (P < 0.05) were significantly elevated in the HHCY group (P < 0.05) (Figures 2A,B). qRT-PCR further confirmed increased mRNA expression of collagen genes (col1α2 and col3α1) in myocardial tissues (P < 0.05) (Figure 2C). Consistently, Masson staining revealed a marked increase in myocardial fibrosis area in the HHCY group (Figure 2D). These findings collectively confirm that HHCY induces myocardial remodeling in SD rats.

Previous studies have reported an anti-myocardial fibrosis role of H_2_S in the myocardium. Our preliminary GEO database analysis (GSE30308) also suggested that HHCY-induced myocardial remodeling is closely associated with mitochondrial dysfunction (Supplementary Figure S1). Therefore, AP39—a mitochondria-targeted H_2_S donor—was selected for therapeutic intervention. HHCY model rats were randomly assigned to the HHCY group and HHCY + AP39 group. Compared with the HHCY group, AP39 treatment significantly increased LVFS (P < 0.05) and decreased LVESD (P < 0.05). Importantly, AP39 alone did not affect cardiac function in normal rats, with no significant changes observed in LVEDD, LVESD, or LVFS (P > 0.05) (Table 3; Figure 2E). At the molecular level, AP39 significantly attenuated HHCY-induced upregulation of α-SMA and Collagen III proteins (P < 0.05) (Figures 2A,B). qRT-PCR results also showed decreased expression of col1α2 and col3α1 mRNA in the HHCY + AP39 group (P < 0.05) (Figure 2C); Masson staining further confirmed that AP39 reduced myocardial fibrotic area in HHCY rats (Figure 2D). Notably, co-administration of the H_2_S synthesis inhibitor PAG (40 mg/kg for 4 weeks) markedly abolished the protective effect of AP39. Taken together, these results indicate that AP39 effectively inhibits HHCY-induced myocardial remodeling by H_2_S, without exerting adverse effects on normal cardiac function.

### 3.3 AP39 inhibits cardiomyocyte senescence under HHCY conditions

**FIGURE 3:**
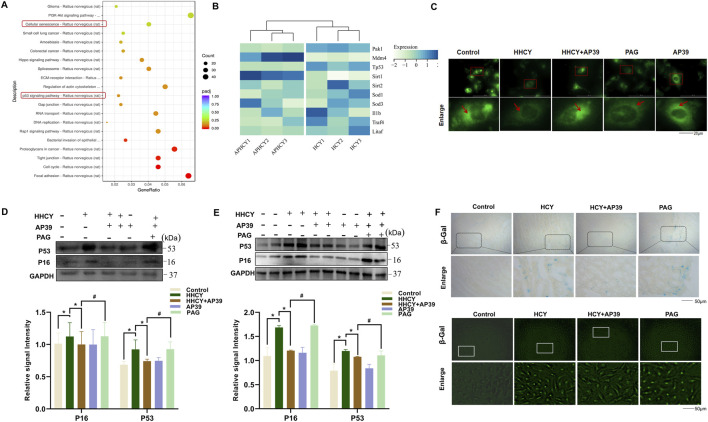
**(A)** KEGG functional enrichment analysis of differentially expressed genes in heart tissues with HHCY-induced myocardial remodeling after AP39 intervention. **(B)** The figure shows the expression of myocardial cell senescence-related genes after AP39 intervention. **(C)** Fluorescence microscopy observation of the changes in mitochondrial H_2_S after AP39 treatment. **(D)** The expression of cellular senescence-related proteins P53 and P16 in heart tissues of each group; **(E)** The expression of cellular senescence-related proteins P53 and P16 in cardiomyocytes of each group. (*p < 0.05 vs. Control; ^#^p < 0.05 vs. HHCY; ^$^p < 0.05 vs. HHCY + AP39). **(F)** Detection of cellular senescence in heart tissues of each group (upper panel) and in cardiomyocytes of each group (lower panel) by β-galactosidase assay. Kyoto Encyclopedia of Genes and Genomes: KEGG; hydrogen sulfide: H_2_S; high homocysteine: HHCY.

The role of cardiomyocytes in the development of myocardial fibrosis has attracted increasing attention (Zhang et al., 2023; Tu et al., 2025). To elucidate how AP39 alleviates HHCY-induced cardiac remodeling, we performed RNA-seq on HHCY-treated cardiomyocytes with AP39 intervention. A total of 1,638 genes were significantly dysregulated, including 897 upregulated and 741 downregulated genes (Supplementary Figure S2). Kyoto Encyclopedia of Genes and Genomes (KEGG) analysis revealed significant enrichment in the cellular senescence g pathway (Figure 3A). Notably, KEGG pathway analysis demonstrated that AP39 intervention specifically upregulated the expression of beneficial genes associated with cellular senescence inhibition, while concurrently downregulating those genes that promote cellular senescence progression (Figure 3B). To further verify the regulatory effect of the H_2_S donor AP39 on the senescence-related proteins P53 and P16, H9c2 cardiomyocytes were first exposed to different concentrations of HCY (0, 0.1 mmol/L, 1 mmol/L, and 2 mmol/L). After incubation for the indicated time, cell viability was evaluated using the CCK-8 assay, and the expression levels of the senescence-associated proteins P53 and P16 were analyzed by Western blotting. The results showed that 1 mmol/L HCY significantly upregulated the expression of P53 and P16 compared with the control group (P < 0.05), while cell viability remained largely unaffected at this concentration (Supplementary Figures S3A, B). Therefore, 1 mmol/L HCY was selected for subsequent experiments. Under the condition of 1 mmol/L HCY stimulation, H9c2 cells were treated with increasing concentrations of AP39 (0, 1 nmol/L, 10 nmol/L, 100 nmol/L, and 300 nmol/L) for 24 h. Cell viability and the protein expression of P53 and P16 were then assessed. The results demonstrated that the mitochondria-targeted H_2_S donor AP39 suppressed the expression of P53 and P16 in a concentration-dependent manner within the range of 1–100 nmol/L, with a statistically significant inhibitory effect observed at 100 nmol/L (P < 0.05). Moreover, AP39 at this concentration did not induce detectable cytotoxicity (Supplementary Figures S3C, D). Based on these findings, 100 nmol/L AP39 was selected as the optimal intervention concentration for the subsequent experiments. To determine whether AP39 selectively delivers H_2_S to mitochondria to exert its cardioprotective effects, we used the mitochondrial H_2_S-specific fluorescent probe Mito-HS (provided by Professor Lin Li’s laboratory, Northwest University) to label mitochondrial H_2_S and assess changes in mitochondrial H_2_S levels in H9c2 cardiomyocytes. Fluorescence imaging demonstrated that mitochondrial H_2_S levels increased significantly following AP39 treatment, whereas PAG markedly suppressed mitochondrial H_2_S production (Figure 3C).

Rescue experiments *in vitro* were further conducted to validate the above findings. H9c2 cardiomyocytes were first pretreated with 2 mmol/L PAG for 30 min, followed by incubation with 100 nmol/L AP39 for 24 h, and subsequently exposed to 1 mmol/L HCY for an additional 24 h. The results showed that PAG markedly reversed the AP39-mediated suppression of P53 and P16 expression (Figure 3E). Furthermore, cellular senescence was assessed using immunofluorescence staining of cell climbing slides combined with β-galactosidase staining. Compared with the control group, the HHCY group exhibited a significant increase in the number of senescent H9c2 cardiomyocytes. AP39 treatment substantially attenuated HHCY-induced cellular senescence, whereas co-treatment with PAG abolished this protective effect, leading to increased cellular senescence (Figure 3F- Lower panel). To further confirm the role of CSE in the AP39-mediated regulation of cellular senescence, a CSE-specific knockdown model was established. The results demonstrated that, following CSE knockdown, AP39 failed to suppress the HCY-induced upregulation of P53 and P16. No significant difference was observed between the CSE knockdown group and the PAG-treated group, indicating that CSE silencing and PAG intervention exert comparable effects within this regulatory pathway (Supplementary Figure S4A–D).

Similarly, cardiac tissues were collected from HHCY SD rats to assess myocardial cellular senescence through β-galactosidase staining and WB. Compared with the Control group, the HHCY group demonstrated a significant upregulation of the senescence-related-associated proteins P53 and P16, accompanied by an increased number of β-galactosidase–positive cardiomyocytes. Administration of AP39 markedly attenuated the HHCY-induced upregulation of these senescence markers, whereas co-treatment with PAG (an inhibitor of the endogenous H_2_S-producing enzyme CSE) significantly blocked the antagonistic effect of AP39 (Figures 3D,F-Up panel).

### 3.4 AP39 inhibits cardiomyocyte senescence and myocardial remodeling by regulating FUNDC1-Mediated mitochondrial dynamics

**FIGURE 4:**
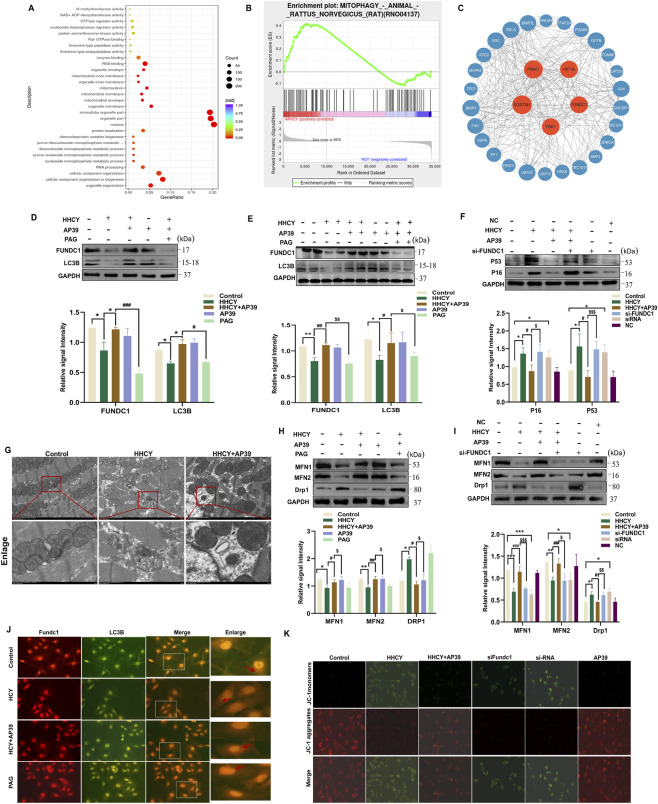
**(A)** GO analysis revealed that cardiomyocytes under HHCY conditions were mainly enriched in mitochondrial processes, mitochondrial function, and inner mitochondrial membrane signaling pathways. **(B)** GSEA of AP39-intervened samples that showed significant enrichment and upregulation of the mitophagy pathway. **(C)** Cystoscope analysis the top 5 ranked genes were SQSTM1, PINK1, HIF1α, FUNDC1, and TBK1. Expression of FUNDC1, LC3A/B in SD rats induced by HHCY **(D)** and H9c2 cardiomyocytes **(E)** in each group. (*p < 0.05 vs. Control; **p < 0.01 vs. Control; #p < 0.05 vs. HHCY; ##p < 0.01 vs. HHCY; $p < 0.05 vs. HHCY + AP39, $$$p < 0.005 vs. HHCY + AP39); **(F)** Expression of P53,P16 in H9c2 cardiomyocytes in each group after siRNA interference. (*p < 0.05 vs. Control; #p < 0.05 vs. HHCY; $$$p < 0.005 vs. HHCY + AP39); **(G)** Observation of changes in Mitophagy formation in each group under transmission electron microscopy. Expression of MFN1, MFN2, Drp1 in SD rats induced by HHCY **(H)** and H9c2 cardiomyocytes **(I)** in each group. (*p < 0.05 vs. Control; **p < 0.01 vs. Control; ***p < 0.005 vs. Control; #p < 0.05 vs. HHCY; ##p < 0.01 vs. HHCY; ###p < 0.005 vs. HHCY; $p < 0.05 vs. HHCY + AP39, $$p < 0.01 vs. HHCY + AP39, $$$p < 0.005 vs. HHCY + AP39); **(J)** Co-localization of FUNDC1 and LC3B in cardiomyocytes in each group under fluorescence microscopy. **(K)** Mitochondrial membrane potential changes after siRNA interference. (Red fluorescence indicates high mitochondrial membrane potential; green fluorescence indicates low mitochondrial membrane potential). Gene Ontology: GO; high homocysteine: HHCY; Gene Set Enrichment Analysis: GSEA.

To elucidate the molecular mechanism by which AP39 inhibits cardiomyocyte senescence, Gene Ontology (GO) analysis revealed that HHCY-treated cardiomyocytes were predominantly enriched in mitochondrial processes, mitochondrial function, and inner mitochondrial membrane signaling pathways (Figure 4A). Gene Set Enrichment Analysis (GSEA) further demonstrated enrichment of the mitophagy pathway following AP39 intervention (Figure 4B). Cystoscope analysis of mitophagy-related genes, Based on Degree Centrality (DC) scores, identified the top five candidates: SQSTM1, PINK1, HIF1α, FUNDC1, and TBK1 (Figure 4C). Integrating GO analysis, PINK1 and FUNDC1 were identified as mitochondrial membrane-related genes. Further verification showed that FUNDC1 exhibited statistical significance in HHCY-induced H9c2 cardiomyocytes, while PINK1 did not (Supplementary Figure S5). To investigate whether AP39 regulates FUNDC1-mediated mitophagy, mitophagy-related proteins was assessed in cardiac tissues from HHCY-induced SD rats using WB. Compared with the Control group, the HHCY group exhibited downregulated of FUNDC1 and LC3A/B which was significantly reversed by AP39. Notably, co-administration of PAG (an inhibitor of the endogenous H_2_S-producing enzyme CSE) blocked the above effects of AP39 (Figure 4D).

In in vitro experiments, H9c2 cardiomyocytes at50%–60% confluency was treated with HCY (1 mM) for 24 h. WB analysis revealed that HHCY significantly suppressed the expression of the mitophagy-related proteins FUNDC1 and LC3A/B, indicating impaired mitophagy, whereas AP39 treatment markedly restored their expression (indicating activated mitophagy). Pretreatment with the H_2_S synthesis inhibitor PAG (2 mmol/L) for 30 min prior to AP39 (100 nmol/L) abrogated the effect of AP39 on FUNDC1 and LC3A/B, with statistical significance (p < 0.05) (Figure 4E). Immunofluorescence analysis demonstrated that AP39 enhanced the colocalization of FUNDC1 (a mitophagy marker protein) and LC3B in cardiomyocytes (Figure 4J), further confirming that AP39 activates FUNDC1-mediated mitophagy.

To further investigate whether FUNDC1-dependent mitophagy mediates anti-senescence effects of AP39, siRNA interference was employed. Three synthetic FUNDC1-siRNA sequences were transfected into well-grown H9c2 cardiomyocytes using Lipofectamine 3000. WB analysis was performed to evaluate FUNDC1 knockdown efficiency. The siRNA demonstrating the highest interference efficiency was selected for subsequent experiments (Supplementary Figure S6). FUNDC1-siRNA was then transfected into H9c2 cardiomyocytes under AP39 treatment, and the expression of senescence-related proteins P53 and P16 was assessed. Results revealed that FUNDC1 knockdown significantly reversed the AP39-mediated downregulation of P53 and P16 in HHCY-induced H9c2 cardiomyocytes (Figure 4F), indicating that AP39 mitigates HHCY-induced cardiomyocyte senescence through FUNDC1-mediated mitophagy. Next, mitochondrial morphology was examined in HHCY-induced myocardial remodeling using electron microscopy. Compared with the Control group, the HHCY group exhibited reduced mitophagosome formation, fragmented mitochondrial cristae, intramitochondrial vacuolization, and disorganized mitochondrial arrangement. AP39 treatment markedly ameliorated these mitochondrial abnormalities (Figure 4G), suggesting that AP39 may also counteract HHCY-induced cardiomyocyte senescence and myocardial remodeling by restoring mitochondrial dynamics. Accordingly, rat cardiac tissues were isolated, and the expression of mitochondrial dynamics-related proteins was assessed by WB. Compared with the Control group, HHCY treatment downregulated the expression of mitochondrial fusion-related proteins MFN1 and MFN2, while upregulated the mitochondrial fission protein DRP1. AP39 intervention markedly restored these protein changes, whereas co-administration of PAG abolished the effects of AP39 (Figure 4H). In in vitro experiments, H9c2 cardiomyocytes were divided into the following groups: Control, HHCY, HHCY + AP39, HHCY + AP39+ FUNDC1-siRNA, FUNDC1-siRNA, NC. Compared with the Control group, the HHCY group significantly upregulated DRP1 and downregulated MFN1/MFN2 (P < 0.05). AP39 intervention reversed these changes, with decreased DRP1 and increased MFN1/MFN2 levels (P < 0.05). No significant differences in DRP1, MFN1, or MFN2 were observed between the Control and NC groups Furthermore, FUNDC1-siRNA markedly abrogated the AP39-mediated regulation of DRP1, MFN1, and MFN2 in HHCY-induced H9c2 cardiomyocytes (Figure 4I). WB analysis of the mitochondrial marker Tom20 showed no significant changes in total mitochondrial content under FUNDC1-siRNA treatment, indicating that the observed effects on mitochondrial dynamics proteins were independent of mitochondrial mass (Supplementary Figure S7).

Mitochondrial membrane potential was assessed using the JC-1 assay, revealing that FUNDC1-siRNA significantly inhibited the AP39- induced restoration of mitochondrial membrane potential in HHCY-treated cells (Figure 4K). Collectively, these results demonstrate that FUNDC1-siRNA effectively reverses the protective effect of AP39 on mitochondrial dynamic homeostasis in HHCY-induced cardiomyocytes. Highlighting FUNDC1 as a critical mediator linking mitophagy and mitochondrial dynamics regulation.

### 3.5 AP39 May inhibit cardiomyocyte senescence by promoting the dissociation of FUNDC1 from DRP1

**FIGURE 5:**
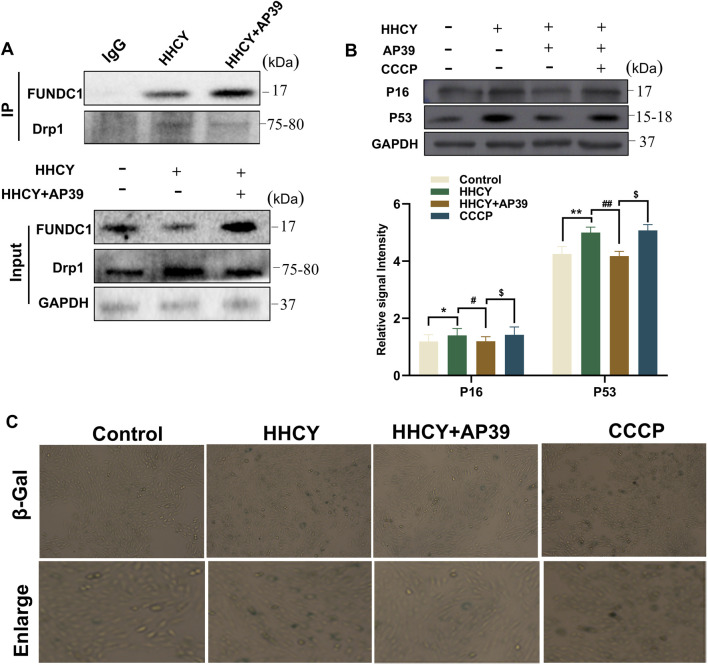
**(A)** Co-Immunoprecipitation (Co-IP) analysis showing the binding of FUNDC1 to DRP1 in cardiomyocytes of each group; **(B)** Expression of P53, P16 in H9c2 cardiomyocytes in each group after siRNA interference. (*p < 0.05 vs. Control; **p < 0.01 vs. Control; #p < 0.05 vs. HHCY; ##p < 0.01 vs. HHCY; $p < 0.05 vs. HHCY + AP39); **(C)** Detection of cellular senescence in heart tissues of each group (upper panel) and in cardiomyocytes of each group (lower panel) by β-galactosidase assay.

A substantial body of evidence suggests that FUNDC1 regulates mitochondrial dynamics by dissociating from DRP1. However, whether FUNDC1 interacts with DRP1 under HHCY-induced cardiomyocyte conditions remains unclear. To investigate this, co-immunoprecipitation (Co-IP) assays were performed, revealing that HHCY treatment significantly increased the binding of FUNDC1 to DRP1 in H9c2 cardiomyocytes, whereas AP39 intervention markedly reduced this interaction (Figure 5A). To further evaluate the functional relevance of FUNDC1-DRP1 dissociation, we established four experimental groups: Control, HHCY, HHCY + AP39, and HHCY + AP39+CCCP (a DRP1 agonist). Compared with the HHCY + AP39 group, cells in the HHCY + AP39+CCCP group exhibited significantly upregulated expression of senescence-related proteins P53 and P16 (Figure 5B). Consistently, β-galactosidase staining demonstrated increased cellular senescence in the HHCY + AP39+CCCP group (Figure 5C). Collectively, these findings indicate that FUNDC1, as a pivotal regulator linking mitophagy and mitochondrial dynamics, modulates mitochondrial fusion-fission balance and cardiomyocyte senescence through competitive binding with DRP1.

### 3.6 AP39 upregulates FUNDC1 expression by inhibiting the NEDD8-CULLIN pathway

**FIGURE 6:**
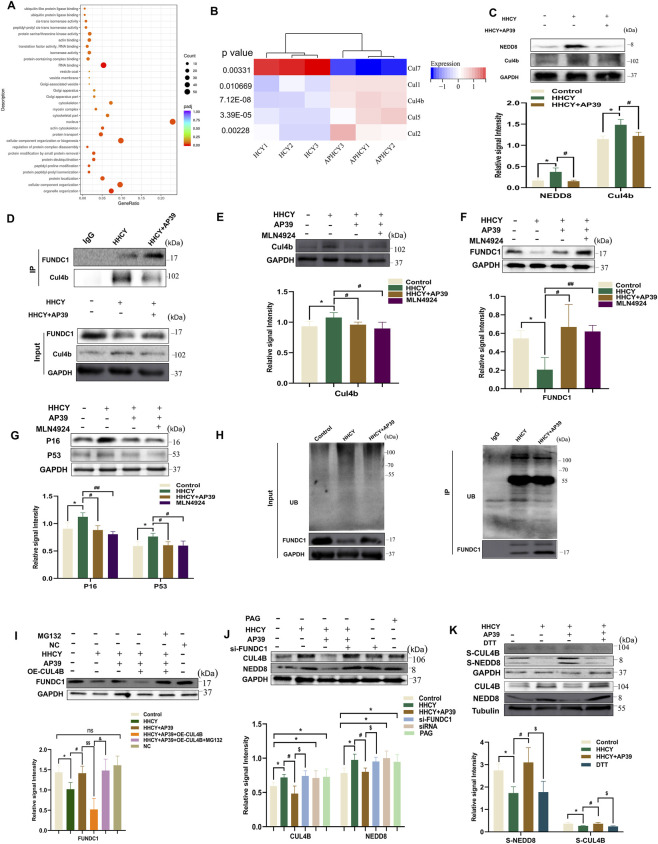
**(A)** KEGG analysis revealed that cardiomyocytes under HHCY conditions were mainly enriched in ubiquitination pathway. **(B)** Heatmap showing differentially expressed CULLIN family genes in HHCY hearts. P value is shown. **(C)** Expression of NEDD8, Cul4b in SD rats induced by HHCY in each group. (*p < 0.05 vs. Control; #p < 0.05 vs. HHCY); **(D)** Co-Immunoprecipitation (Co-IP) analysis showing the binding of FUNDC1 to Cul4b in cardiomyocytes of each group; **(E–G)** Expression of Cul4b, FUNDC1, P53, P16 in H9c2 cardiomyocytes in each group. (*p < 0.05 vs. Control; #p < 0.05 vs. HHCY; ##p < 0.01 vs. HHCY); **(H)** Co-immunoprecipitation (Co - IP) and ubiquitination assays were performed to assess FUNDC1 ubiquitination in Control, HHCY, and HHCY + AP39 groups. **(I,J)** Expression of FUNDC1, NEDD8, CUL48in H9c2 cardiomyocytes in each group. **(K)** Expression of NEDD8, CUL48, S-NEDD8, S-CUL4B in H9c2 cardiomyocytes in each group. (*p < 0.05 vs. Control; #p < 0.05 vs. HHCY; $p < 0.05 vs. HHCY + AP39).

To further elucidate how AP39 regulates FUNDC1 expression. RNA-seq analysis was performed in HHCY-induced H9c2 cardiomyocytes treated with AP39 intervention, KEGG pathway enrichment indicated that the ubiquitination pathway was significantly enriched among the genes improved by AP39 under HHCY conditions (Figure 6A). Screening of all ubiquitination-related genes with statistical significance revealed that that CUL4B displayed the most prominent differential expression (Figure 6B). As a core scaffold protein of the CULLIN family, CUL4B initiates the ubiquitin-dependent degradation process through binding to NEDD8, and is closely associated with mitochondria. These findings led us to hypothesize that AP39 may enhance FUNDC1 expression by inhibiting the NEDD8–CUL4B pathway.

First, we detected that the expression of NEDD8 and CUL4B were markedly upregulated in cardiac tissues from HHCY-induced rats with myocardial remodeling, whereas AP39 intervention downregulated both proteins (Figure 6C). We subsequently validated the physical interaction between CUL4B and FUNDC1 through Co-IP experiments, and observed that AP39 treatment notably weakened this interaction, indicating reduced CUL4B-mediated regulation of FUNDC1 (Figure 6D).

In addition, four experimental groups were established: Control (CON), HHCY model, HHCY + NEDD8 inhibitor, and AP39 intervention. Compared with the Control group, the HHCY group exhibited significantly decreased FUNDC1 expression and increased levels of CUL4B, P53, and P16 (P < 0.05). Both the HHCY + AP39 group and the HHCY + NEDD8 inhibitor group showed markedly upregulated FUNDC1 and downregulated CUL4B, P53, and P16 relative to the HHCY group (P < 0.05). No significant differences in the expression of CUL4B, FUNDC1, P53, or P16 expression were observed between the Control and AP39 groups (Figures 6E–G). These results indicate that the AP39 and the NEDD8 inhibitor exert comparable regulatory effects on regulating FUNDC1 expression. Furthermore, Co-IP analysis of FUNDC1 ubiquitination revealed that the HHCY group displayed increased ubiquitination of FUNDC1, whereas AP39 intervention markedly reduced this modification -further supporting that AP39 suppresses FUNDC1 ubiquitination by inhibiting the NEDD8-CUL4B pathway (Figure 6H). To clarify the upstream–downstream regulatory relationship between NEDD8/CUL4B and FUNDC1, we established a CUL4B overexpression model and a FUNDC1 knockdown model, respectively. Western blot analysis showed that, compared with the HCY + AP39 group, FUNDC1 protein levels were significantly reduced in the HCY + AP39+CUL4B-overexpression group (Figure 6I). In contrast, in the FUNDC1-knockdown group (HCY + AP39+siRNA-FUNDC1), the protein expression of NEDD8 and CUL4B showed no obvious changes. These results indicate that NEDD8/CUL4B acts as an upstream regulator that negatively modulates FUNDC1 expression, whereas FUNDC1 does not exert feedback regulation on NEDD8/CUL4B (Figure 6J). To further investigate the specific mechanism by which NEDD8/CUL4B regulates FUNDC1, we included a pretreatment group with the proteasome inhibitor MG132 (Figure 6K). The results revealed that FUNDC1 protein levels in the HCY + AP39 + CUL4B-overexpression + MG132 group were significantly restored compared with the HCY + AP39+CUL4B-overexpression group, reaching a level close to that of the HCY + AP39 group. This “rescue experiment” confirms that the negative regulation of FUNDC1 by NEDD8/CUL4B depends on the ubiquitin–proteasome degradation pathway.

In recent years, accumulating evidence has demonstrated that hydrogen sulfide (H_2_S) exerts many of its biological effects through protein S-sulfhydration. S-sulfhydration is a novel H_2_S-mediated post-translational modification inwhich a persulfide groups (-SSH) is added to specific cysteine residues, converting the thiol group (-SH) into a persulfide moiety and thereby altering protein conformation, activity, and downstream signaling. Importantly, this covalent modification is reversible, as strong reducing agents such as DTT can remove the persulfide group and restore the original thiol state (Wu et al., 2018; Bibli et al., 2019; Meng et al., 2018; Cheung and Lau, 2018). Thus, we hypothesized: does the inhibition of the NEDD8-CUL4B pathway by AP39 involve S-sulfhydration modification of NEDD8? By reviewing the literature, we found that a previous S-sulfhydration mass spectrometry analysis identified S-sulfhydration modification of NEDD8. To validate this hypothesis, we first assessed the S-sulfhydration levels of key molecules using a modified biotin-switch assay. Four groups were established: CON, HHCY, HHCY + AP39, and HHCY + AP39+DTT (with DTT applied to reverse S-sulfhydration). The results showed that NEDD8 and CUL4B exhibited markedly reduced S-sulfhydration in the HHCY group compared with the Control group, indicating that HHCY suppresses S-sulfhydration within this regulatory axis. In contrast, AP39 significantly restored the S-sulfhydration levels of both NEDD8 and CUL4B relative to the HHCY group, confirming that AP39 promotes their S-sulfhydration. Importantly, DTT completely abolished the AP39-induced enhancement of NEDD8 and CUL4B S-sulfhydration (Figure 6K), demonstrating that this regulatory effect is specifically dependent on S-sulfhydration modification. Furthermore, through the construction of a CUL4B overexpression model, we demonstrated that CUL4B overexpression significantly counteracted the ameliorative effect of AP39 on cellular senescence, confirming that the NEDD8/CUL4B axis is a central pathway through which AP39 exerts its anti-aging effects (Figure 6M).

Taken together, these findings demonstrate that AP39 enhances the S-sulfhydration of both NEDD8 and CUL4B, thereby suppressing the activity of the NEDD8/CUL4B pathway. This inhibition consequently reduces the ubiquitin-mediated degradation of FUNDC1, ultimately leading to upregulated FUNDC1 expression.

## 4 Discussion

This study systematically elucidated the molecular mechanisms underlying HHCY-induced myocardial remodeling and identified the therapeutic effects and regulatory pathways of the mitochondria-targeted H_2_S donor AP39. By integrating clinical evidence with animal experiments and cellular and molecular analyses, our findings demonstrate that HHCY promotes myocardial remodeling primarily through the induction of cardiomyocyte senescence and disruption of mitochondrial dynamic homeostasis. In contrast, AP39 exerts significant cardioprotective effects by restoring FUNDC1-mediated mitophagy, while concurrently suppressing the activation of the NEDD8-CUL4B pathway.

H_2_S, recognized as the third gas transmitter, exerts well-established anti-inflammatory and cytoprotective effects, and has been reported to possess anti-myocardial fibrosis properties (Peake et al., 2013). In the present study, clinical data analysis first revealed a dose-dependent positive correlation between serum HCY levels and the incidence of left ventricular hypertrophy (LVH). Receiver operating characteristic (ROC) curve analysis further demonstrated the predictive value of HCY for LVH (AUC = 0.737), thereby providing robust clinical evidence to support subsequent mechanistic investigations. To further validate the pathological role of HHCY, an HHCY rat model was established via L-methionine administration. Functional and structural assessments revealed impaired LVSF, characterized by reduced LVFS and LVESD, accompanied by a marked upregulation of myocardial fibrosis-related markers, includingα-SMA, Collagen III, col1α2, col3α1. These findings collectively confirm that HHCY can directly induce myocardial remodeling. Notably, treatment with the mitochondria-targeted H_2_S donor AP39 significantly alleviated HHCY-induced cardiac dysfunction and myocardial fibrosis. Importantly, these protective effects were largely abolished by the H_2_S synthesis inhibitor PAG, indicating that the anti-remodeling actions of AP39 are dependent on its H_2_S-releasing capacity. Collectively, this result not only extend the cardioprotective role of H_2_S in the context of HHCY-induced myocardial remodeling, but also underscore the therapeutic advantage of mitochondrial-targeted H_2_S delivery in preserving cardiac structure and function.

Accumulating evidence indicates that cellular senescence plays a pivotal role in the initiation and progression of myocardial remodeling. Moreover, multiple studies have demonstrated that HHCY is a inducer of cellular senescence (Kwak et al., 2015; Sun et al., 2019), suggesting that targeting senescence may represent an effective therapeutic strategy for HHCY-induced myocardial remodeling. In the present study, senescence-associated β-galactosidase staining and immunofluorescence assays, together with Western blot analysis of senescence-related markers (P53, P16), consistently confirmed that HHCY robustly induces senescence in H9c2 cardiomyocytes. Notably, AP39 treatment markedly attenuated these senescence-associated phenotypes, indicating that the cardioprotective effect of AP39 are at least partially mediated through the suppression of cardiomyocyte senescence. Collectively, these findings finding provide novel cellular-level evidence linking HHCY-induced myocardial remodeling to cardiomyocyte senescence and offer a more precise mechanistic explanation for the therapeutic efficacy of AP39, thereby expanding our understanding of the senescence-centered regulatory network underlying HHCY-related cardiac remodeling.

Cellular senescence can be triggered by oxidative stress, lipotoxicity, telomere shortening, metabolic disorders, and DNA damage. To gain deeper insights into the mechanism of HHCY-induced cardiomyocyte senescence, we conducted comprehensive RNA-seq analysis, which identified FUNDC1 as a potential key molecule in cardiomyocyte senescence. Notably, our RNA-seq data revealed no significant differences in PINK1 gene expression across experimental groups, suggesting that under these specific conditions, the protective effects of AP39 are independent of transcriptional activation of the PINK1-Parkin axis. Further *in vivo* and *in vitro* experiments confirmed that FUNDC1 was significantly downregulated in the HHCY group, and this downregulation was reversed by AP39 intervention. On this basis, cellular interference experiments showed that silencing FUNDC1 significantly upregulated the expression of senescence-related proteins P53 and P16, indicating that AP39 may inhibit cardiomyocyte senescence through FUNDC1. Future studies could explore potential crosstalk or context-dependent hierarchy between these pathways under different stresses.

We further demonstrated that the correction of mitochondrial dynamic imbalance represents a critical mechanism by which AP39 suppresses cardiomyocyte senescence in a FUNDC1-dependent manner. Emerging evidence indicates that alterations in mitochondrial dynamics are intimately involved in the initiation and progression of cellular senescence (Camacho-Encina et al., 2024), with the coordinated balance between mitochondrial fission and fusion being essential for maintaining mitochondria homeostasis. In the present study, both *in vitro* and *in vivo* experiments under HHCY stimulation revealed a pronounced disruption of mitochondrial dynamics, characterized by a significant upregulation of the mitochondrial fission protein DRP1, concomitant downregulated of mitochondrial fusion proteins MFN1 and MFN2, and a marked reduction in mitochondrial membrane potential. Notably, these pathological alterations were effectively reversed following AP39 treatment, concomitant with the upregulation of FUNDC1. In contrast, silencing FUNDC1 in the presence of AP39 abolished these protective effects, leading to reactivation of DRP1 and suppression of MFN1/MFN2 expression.

These findings provide compelling evidence that the restoration of mitochondrial dynamic homeostasis is indispensable for AP39-mediated inhibition of cardiomyocyte senescence and that this process critically depends on FUNDC1 signaling. Previous studies have reported that under hypoxic or stress-related conditions, FUNDC1 can preferentially recruit DNM1L/DRP1 to mitochondria, thereby promoting mitochondrial fission and triggering mitochondrial dynamic imbalance (Bai et al., 2023). Consistent with this mechanism, our Co-IP experiments demonstrated a direct interaction between FUNDC1 and DRP1 in cardiomyocytes exposed to HHCY, an interaction that was significantly attenuated following AP39 intervention. Based on these observations, we propose that AP39 modulates mitochondrial dynamics by limiting the pathological interaction between FUNDC1 and DRP1 under HHCY conditions, thereby preserving mitochondrial integrity and ultimately inhibiting cardiomyocyte senescence.

To elucidate the upstream molecular mechanism by which AP39 upregulates FUNDC1, transcriptomic enrichment analysis revealed that the ubiquitin–proteasome pathway was significantly enriched following AP39 intervention. Among the core components of this pathway, Cullin 4B (CUL4B), CUL4B a central scaffold protein of E3 ubiquitin ligase complexes, was identified as a direct binding partner of FUNDC1, as confirmed by Co-IP assays. To further define the regulatory relationship among AP39, the NEDD8/CUL4B axis, and FUNDC1, four groups were established: Control, HHCY, HHCY + AP39, and HHCY + MLN429 (a NEDD8 inhibitor). Notably, AP39 treatment phenocopied the effects of NEDD8 inhibition, leading to a marked upregulation of FUNDC1 expression. Mechanistically, both AP39 and MLN4294 suppressed activation of the NEDD8/CUL4B pathway, thereby preventing FUNDC1 downregulation and subsequently restoring mitochondrial dynamic balance and attenuating cardiomyocyte senescence. These findings identify the NEDD8/CUL4B–FUNDC1 signaling axis as previously unrecognized regulatory pathway underlying the cardioprotective effects of AP39 in HHCY-induced myocardial remodeling. To determine whether the NEDD8/CUL4B axis regulates FUNDC1 through ubiquitin-mediated proteasomal degradation, we further established the following experimental groups: Control, HHCY, HHCY + AP39, HHCY + AP39+CUL4B overexpression, and HHCY + AP39+CUL4B overexpression combined with the proteasome inhibitor MG132. The results demonstrated that CUL4B overexpression markedly reduced FUNDC1 protein levels despite AP39 treatment, whereas co-administration of the ubiquitination/proteasome inhibitor MG132 effectively rescued FUNDC1 expression. These findings provide direct mechanistic evidence that AP39 exerts its anti-senescent and anti-fibrotic effects by inhibiting NEDD8/CUL4B–mediated ubiquitination and proteasomal degradation of FUNDC1, thereby preserving mitochondrial homeostasis and attenuating HHCY-induced myocardial remodeling.

As demonstrated by previous studies, the cardioprotective effects of AP39 are primarily mediated through H_2_S-mediated S-sulfhydration. Accordingly, we introduced the S-sulfhydration inhibitor dithiothreitol (DTT) into an AP39-treated, HHCY-induced cardiomyocyte model to assess its impact on cellular senescence. The re-emergence of senescent phenotypes following DTT treatment indicates that AP39 on cardiomyocyte senescence under HHCY conditions is largely dependent on S-sulfhydration. However, the specific molecular targets of this post-translational modification remained to be determined.

Based on these findings, we further investigated whether AP39 exerts its regulatory effects through S-sulfhydration of the NEDD8/CUL4B axis. Using a modified biotin-switch assay, we assessed the S-sulfhydration levels of NEDD8 and CUL4B. The results showed that S-sulfhydration of both NEDD8 and CUL4B was significantly reduced in the HHCY group, markedly increased following AP39 treatment, and abolished by DTT-mediated inhibition of S-sulfhydration. Furthermore, overexpression of CUL4B led to an increase in FUNDC1 expression, whereas co-treatment with DTT substantially attenuated this effect. Collectively, these findings suggest that AP39 may promote FUNDC1 stabilization by inducing S-sulfhydration of NEDD8/CUL4B, thereby restoring mitochondrial dynamic balance, suppressing cardiomyocyte senescence, and ultimately alleviating HHCY-induced myocardial remodeling.

## 5 Study limitations and future perspectives

This study has several limitations: First, the clinical data were derived from a retrospective analysis; a prospective cohort study is needed to verify the causal relationship between HCY and myocardial remodeling. Second, the specific domains involved in the FUNDC1-DRP1 interaction and *in vitro* verification of AP39-mediated S-sulfhydration modification of NEDD8 require further investigation. Third, it is worth exploring whether the NEDD8-CUL4B pathway is involved in the regulation of other mitochondria-related proteins. Future research can focus on: (1) developing small-molecule drugs targeting FUNDC1; (2) establishing specific detection methods for NEDD8 S-sulfhydration modification; (3) verifying the *in vivo* roles of key molecules; (4)Due to the lack of high-resolution mass spectrometry–based proteomic analysis, the precise S-sulfhydration site(s) on NEDD8 could not be definitively identified. While our data demonstrate that AP39-induced H_2_S signaling modulates the NEDD8/CUL4B axis through S-sulfhydration-dependent mechanisms, it remains unresolved whether this post-translational modification occurs on NEDD8 alone or on the NEDD8/CUL4B complex as a functional unit. Distinguishing between these possibilities will be critical for fully understanding how S-sulfhydration dynamically regulates ubiquitin ligase activity under hyperhomocysteinemic conditions (Annoni et al., 1998). Although pharmacological inhibition of S-sulfhydration using DTT and functional rescue experiments provide strong indirect evidence for the involvement of S-sulfhydration, future studies employing site-directed mutagenesis of NEDD8, in combination with quantitative redox proteomics, will be necessary to establish a direct causal relationship between specific S-sulfhydration events and FUNDC1 stabilization. Addressing these limitations in future work will further refine the molecular resolution of the H_2_S–NEDD8/CUL4B–FUNDC1 signaling axis and strengthen its translational relevance in myocardial remodeling.

## 6 Conclusion

In summary, this study demonstrates that AP39 attenuates HHCY-induced myocardial remodeling through the following pathway: H_2_S released from AP39 induces S-sulfhydration of the NEDD8/CUL4B axis, thereby preventing FUNDC1 downregulation, stabilized FUNDC1 subsequently weakens its interaction with DRP1, restores mitochondrial dynamic homeostasis, and ultimately suppresses cardiomyocyte senescence. This pathway provides a novel therapeutic target and theoretical basis for the treatment of HHCY-related cardiovascular diseases.

## Data Availability

The data presented in the study are deposited in the Figshare repository, DOI: 10.6084/m9.figshare.31385917.
