# Supplementary material for: AP39 alleviates HHCY-induced myocardial remodeling by regulating FUNDC1-mediated mitochondrial dynamics via S-sulfhydration of NEDD8/CUL4B
Source: Front Pharmacol. 2026 Mar 9;17:1729145. doi: 10.3389/fphar.2026.1729145 (PMC13006662; doi:10.3389/fphar.2026.1729145)
Supplement: Supplementary file 1 [file Supplementaryfile1.docx]

**S-Table 1 Correlation Analysis between HCY and IVST、LVEDD、LVPWT、LVEF**

| Cardiac Indexes | Hypertension complicated by HHCY | Hypertension | Z | P |
| --- | --- | --- | --- | --- |
| IVST | 10(9~12) | 10(9~10) | -6.005 | P<0.001 |
| LVEDD | 46(43~50) | 45(43~48) | -3.541 | P<0.001 |
| LVPWT | 9(8~10) | 9(8~9) | -6.462 | P<0.001 |
| LVEF | 63(57~68) | 64(60~68) | -2.589 | 0.010 |

Note: Interventricular Septal Thickness (IVST), Left Ventricular End - Diastolic Diameter (LVEDD), Left Ventricular Posterior Wall Thickness (LVPWT), Left Ventricular Ejection Fraction (LVEF), hyper homocysteinemia (HHCY)

**S-Table 2 After propensity score matching, correlation Analysis between HCY and IVST、LVEDD、LVPWT、LVEF**

| Cardiac Indexes | Hypertension complicated by HHCY | Hypertension | Z | P |
| --- | --- | --- | --- | --- |
| IVST (mm) | 10(9~11) | 10(9~10) | -2.019 | 0.043 |
| LVEDD (mm) | 45.50(42~48) | 45(43~48) | -0.001 | 0.999 |
| LVPWT (mm) | 9(8~10) | 9(8~9) | -1.999 | 0.046 |
| LVEF (%) | 64(64~68) | 64(59.50~68) | -0.369 | 0.712 |

Note: Interventricular Septal Thickness (IVST), Left Ventricular End - Diastolic Diameter (LVEDD), Left Ventricular Posterior Wall Thickness (LVPWT), Left Ventricular Ejection Fraction (LVEF), hyper homocysteinemia (HHCY)

**S-Table 3 The plasma HCY levels in each group（mean± SD）**

| Groups | number | HCY (μ mol/L) |
| --- | --- | --- |
| Control | 10 | 7.33±5.62 |
| HHCY | 8 | 18.73±2.82^*^ |
| HHCY+AP39 | 8 | 18.46±2.86^*^ |
| AP39 | 10 | 4.29±2.75 |

Note: *P<0.05 vs Control group.
