# Supplementary material for: AP39 alleviates HHCY-induced myocardial remodeling by regulating FUNDC1-mediated mitochondrial dynamics via S-sulfhydration of NEDD8/CUL4B
Source: Front Pharmacol. 2026 Mar 9;17:1729145. doi: 10.3389/fphar.2026.1729145 (PMC13006662; doi:10.3389/fphar.2026.1729145)

Supplementary Fig1. Scatter plot of Gene Ontology (GO) enrichment analysis in hearts with hyper homocysteinemia (HHCY)-induced cardiac remodeling.


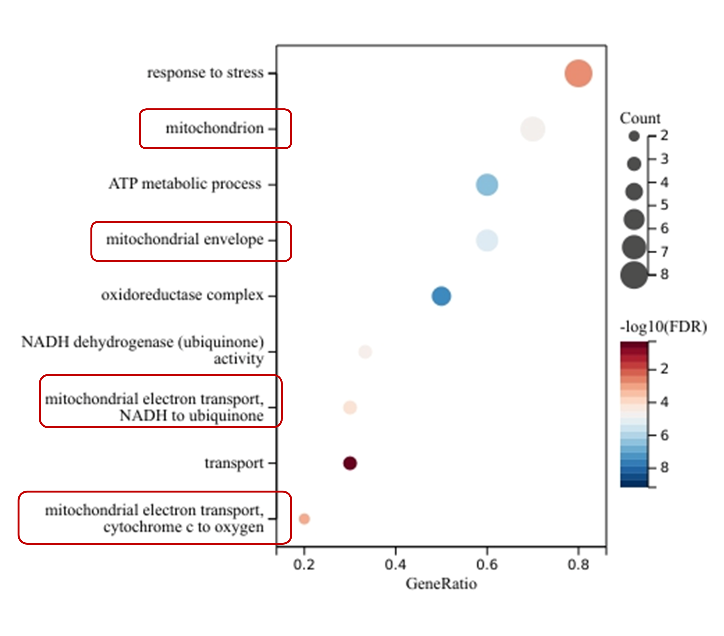


Supplementary Fig2: Differentially expressed genes in RNA sequencing analysis of cardiomyocytes under HHCY condition with AP39 intervention


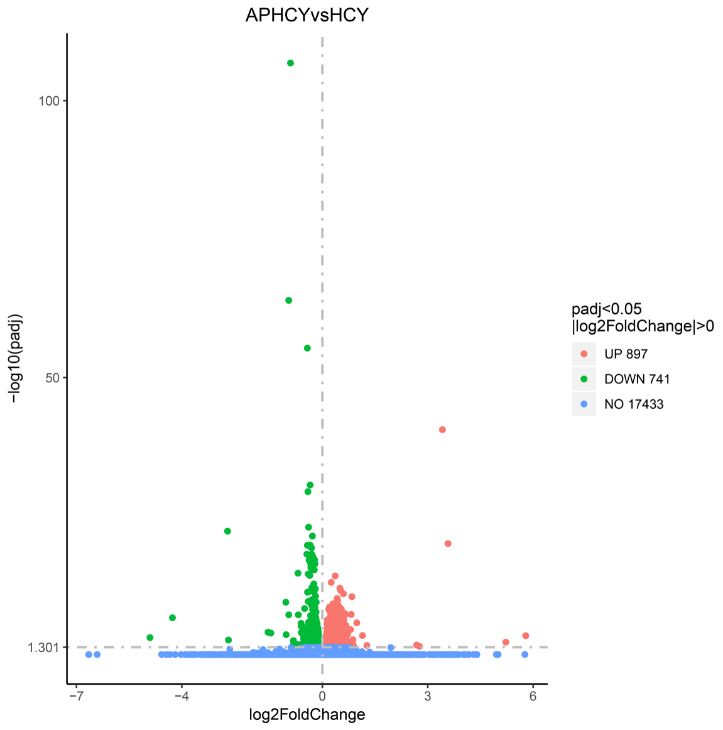


Supplementary Fig3: Detection of the expression of various P53(A, E), P16(B, D) by Western Blot (WB). (C, F) Cell Counting Kit-8 (CCK-8) assay was used to detect the cell viability of each group.


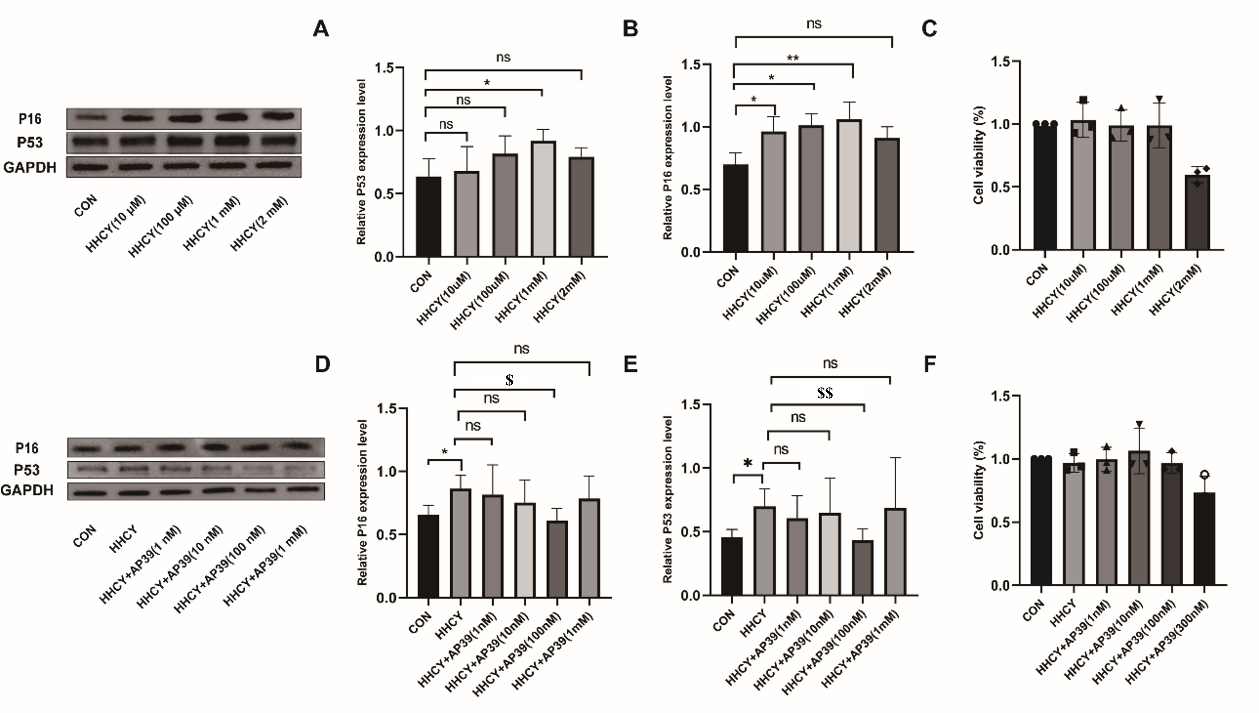


Supplementary Fig4: Detection of the expression of various CSE by Western Blot (WB)(A) and QTPCR(B). Detection of the expression of various P53, P16 by Western Blot (WB). (C, D)


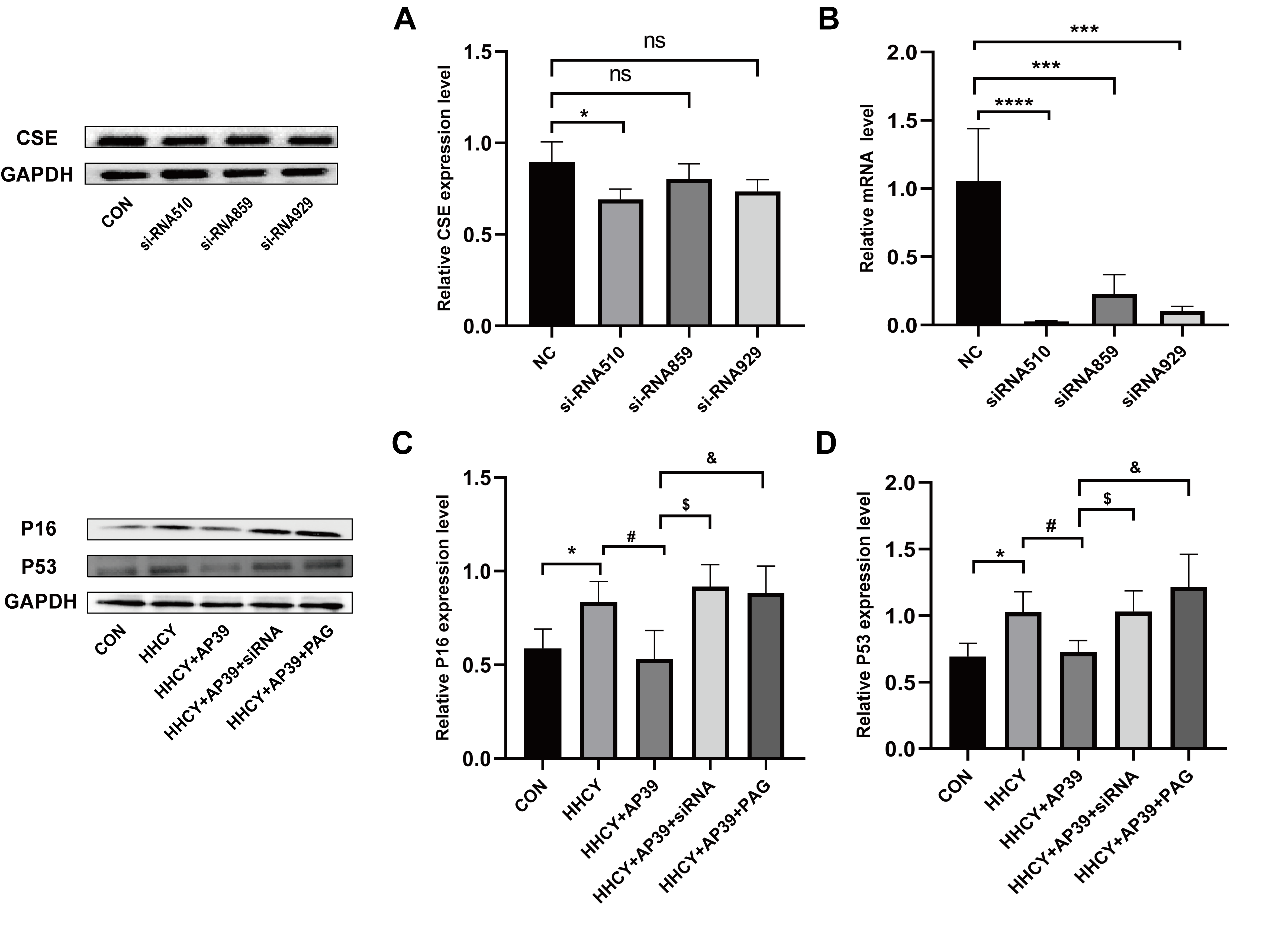


Supplementary Fig5: Heatmap showing differentially expressed FUNDC1, Pink1 in HHCY hearts. P value is shown.


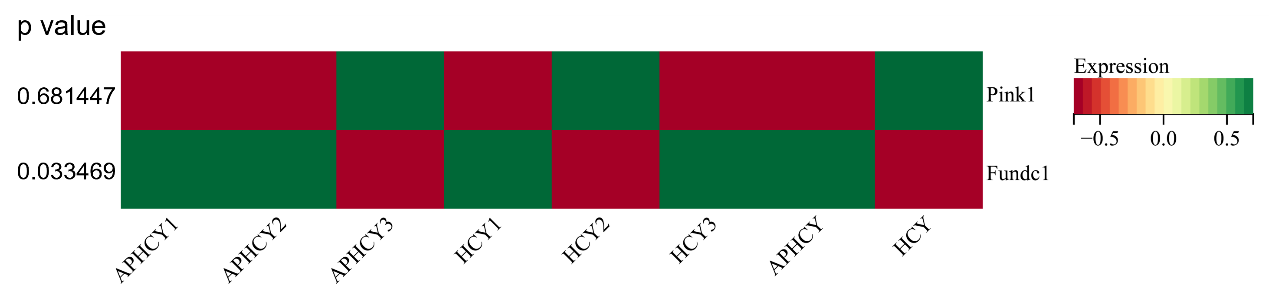


Supplementary Fig6: Detection of FUNDC1 expression by Western Blot (WB); the interfering strand with the optimal knockdown efficiency was selected based on the results for subsequent experiments.
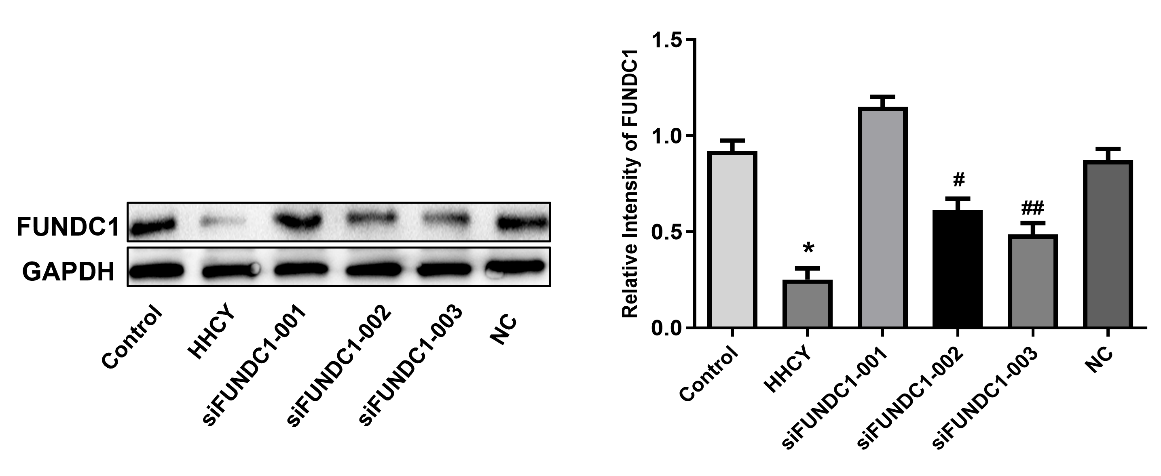


Supplementary Fig7: Detection of TOM20 expression by Western Blot (WB).


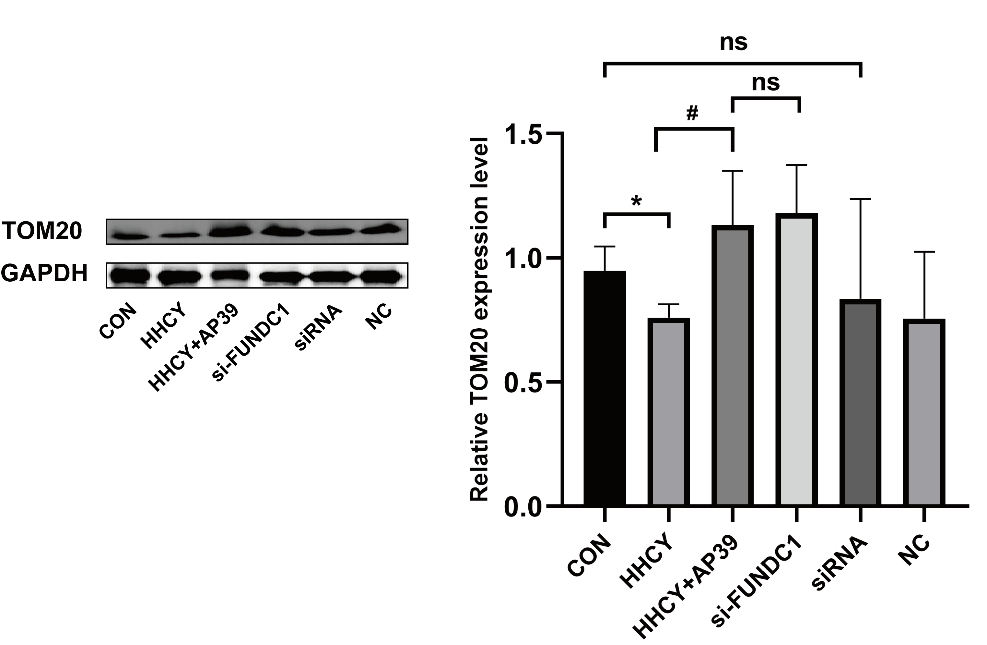

Supplement: Supplementary file 2 [file Supplementaryfile2.docx]
